# Bacterial and Fungal Communities in Old Vines and Their Progeny: Insights into Microbial Inheritance Through Mass Selection

**DOI:** 10.3390/microorganisms14030622

**Published:** 2026-03-10

**Authors:** Solène Lemichez, Maria Bernard, Véronique Chable

**Affiliations:** 1 INRAE, UMR 0908 BAGAP, Domaine de la Motte, 35650 Le Rheu, France; solene.lemichez@inrae.fr; 2 INRAE, AgroParisTech, GABI, Université Paris-Saclay, 78350 Jouy-en-Josas, France; maria.bernard@inrae.fr; 3 INRAE, SIGENAE, 78350 Jouy-en-Josas, France

**Keywords:** grapevine microbiome, mass selection, microbial inheritance, community assembly processes, transdisciplinary research

## Abstract

Mass selection is increasingly promoted in viticulture to enhance resilience by restoring intra-varietal diversity, yet its effects on the structure and inheritance of plant-associated microbiomes remain poorly understood. Here, we investigated bacterial and fungal communities associated with old grapevine mother plants and their progeny across four Bordeaux estates practicing mass selection, using a fully in situ experimental design. Root and leaf microbiomes were characterized by metabarcoding and analyzed using multivariate ordination, hierarchical clustering, and assembly-process metrics (βNTI and NST). Microbial community composition and structure were primarily shaped by plant compartment and vineyard origin, whereas generation effects were significant but weak. Microbial resemblance between mother vines and their offspring was limited and highly context-dependent, occurring mainly under comparable environmental conditions. Assembly-process analyses revealed heterogeneous deterministic signals, particularly in root-associated bacterial communities, but did not consistently result in phylogenetic similarity between generations. Although inheritance signals were generally weak, their recurrence across multiple vineyards and contrasted field conditions highlights their ecological relevance. By integrating environmental variability, this in situ approach mitigates the adaptive bias in plant–microbiome interactions and shows that mass selection does not rely on systematic microbial transmission but rather operates within a nuanced framework of environmentally mediated interactions.

## 1. Introduction

**Figure 1:**
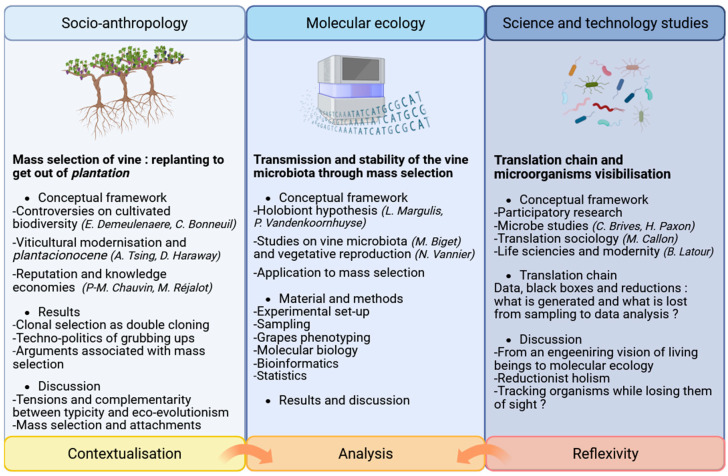
General structure of the project showing the three main steps (contextualization, analysis and reflexivity), their interactions and their objectives.

Viticulture is addressing more and more challenges, including climate change, increased pressure from pests and pathogens, and the erosion of genetic diversity within grapevine cultivars. Clonal selection, mostly used to produce vine plants since the seventies to ensure uniformity and stability of varietal traits, has also contributed to the homogenization of vineyards, reducing their adaptive potential and increasing vulnerability to environmental changes [1]. This context requires a shift in practices: an increasing number of wine estates engage in an ecologisation process, considering among other levers agroecology, agroforestry, crops diversification, and varietal adaptation. In this study, we focus on vine (*Vitis vinifera* subsp. *vinifera* L.) mass selection within the following four estates in the Bordeaux region: Chateau Lafite, Chateau Latour, Chateau Leoville las Cases and Chateau Palmer. In such a participatory project, with a collaborative elaboration of research questions, the diversity of partners implied is often associated with asymmetries of knowledge, expectations and postures. To face this challenge, we discuss the biological hypothesis within a larger framework, not only using molecular ecology in our research but also social sciences (Figure 1).

Over the past decades, advances in eco-evolutionary knowledge and the development of next-generation sequencing techniques have made it possible to study plant–microorganism interactions at increasingly fine scales. The ubiquity of symbiotic relationships in multicellular organisms has led to the emergence of the holobiont hypothesis [2], which considers the host organism (here, the grapevine) and its associated microorganisms as a single, dynamic entity. The involvement of these microorganisms in numerous processes, such as resistance to biotic and abiotic stresses, gives them a crucial role in plant survival, adaptation, and evolution [3]. These findings have contributed to a growing awareness that the grapevine is not merely an isolated plant, but functions as a holobiont: an ecological unit composed of the host and its associated microbial communities. These bacteria and fungi play essential roles in plant health, nutrient acquisition, stress resistance, and ultimately fruit and wine quality [4,5,6]. Understanding how viticultural practices shape grapevine-associated microbiota is therefore key to building sustainable production systems with high-quality objectives.

Recent studies have highlighted multiple drivers of microbiome assembly in grapevines. Genotype, including both scion and rootstock, has strong effects on rhizosphere and root-endophyte communities, with rootstocks often having the greatest influence [7]. Similarly, cultivar identity can shape the leaf microbiome structure, even under similar environments [8]. Environmental and edaphic factors, such as soil properties and vintage, also interact with management practices to influence microbial diversity and composition in vineyards [5,9]. Together, these findings show that grapevine microbiomes are influenced by both host genotype and environmental conditions.

The holobiont paradigm shift has also broadened the concept of heredity, particularly in the context of clonal propagation, by highlighting the potential role of microbial inheritance—the transmission of microbial communities across plant generations. Although the rules governing microbial community assembly are not yet fully understood, increasing evidence suggests that vertical transmission of microbiota may be both more common and more ecologically significant than previously thought. For instance, Vannier et al. (2018) showed that clonal offspring of *Glechoma hederacea* inherits a substantial fraction of its mother plant’s bacterial and fungal symbionts, even when grown under sterile conditions, although with reduced richness compared to the parent, suggesting a process of selective filtering [10]. Similar findings have been reported in other systems, including seed-borne microbiota in oak trees [11], beans and peas [12]. Building on empirical studies, recent conceptual syntheses have argued for a central role of microbial inheritance in shaping plant holobionts and have even proposed viewing clonal plants as meta-holobionts [13].

However, the extent to which microbial communities associated with perennial crops are transmitted during vegetative propagation remains poorly understood, particularly under field conditions. This question is particularly relevant for mass selection in viticulture, where young plants are propagated from older vines but subsequently undergo grafting, nursery cultivation, and transplantation in new environments. Whether microbial communities associated with old vines are conserved, reshaped, or entirely reassembled during this process remains largely unknown.

Our study addresses this gap using an in situ experimental design built upon pre-existing mass selection of four wine estates, where we compared the bacterial and fungal communities in roots and leaves of old vines (PM) and their descendants (PF). We hypothesize the following:

**H1.**  *Generation (mother or daughter vine) influences microbial community structure and composition.*

**H2.**  *Daughter vines host microbial communities that are more phylogenetically similar to their corresponding mother vine than to unrelated vines.*

**H3.**  *The assembly of microbial communities between mother vines and their offspring is primarily driven by deterministic processes.*

By situating microbial ecology research within the real-world of viticultural practice, our aim is to contribute to a broader understanding of how mass selection may preserve not only genetic, but also microbial diversity, thus reinforcing the sustainability of grapevine cultivation.

## 2. Materials and Methods

### 2.1. Experimental Set-Up

**Figure 2:**
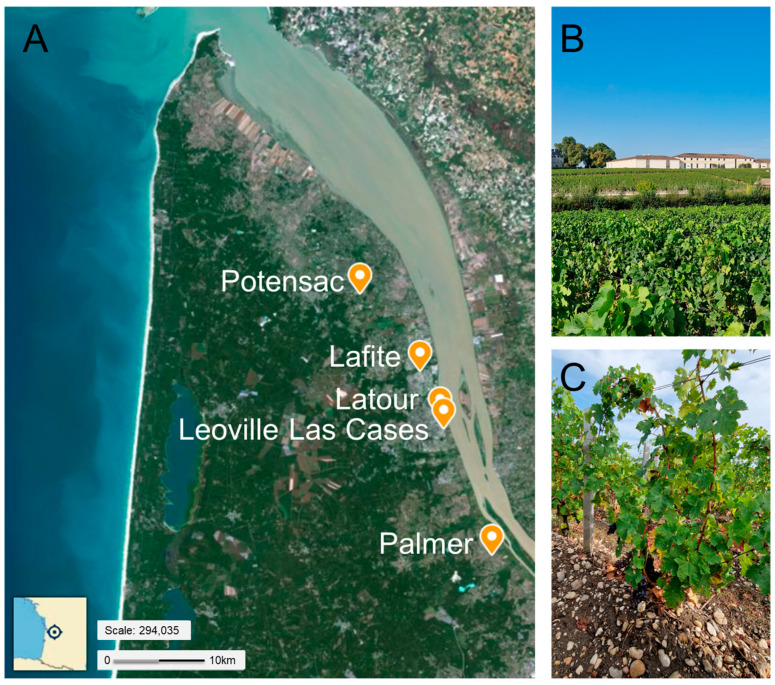
Study sites and context of the Medoc region, France. (**A**) Location of the sampling sites. Potensac corresponds to the plot where young vines from Chateau Leoville las Cases are implanted. (**B**) General view of a vine plot in September 2024. (**C**) Example of a sampled young vine.

Our study sites are located in four wine estates of the Medoc region, covering three appellations: Pauillac (Chateau Latour and Chateau Lafite), Saint-Julien (Chateau Leoville Las Cases), and Margaux (Chateau Palmer). The four estates belong to the same bioclimatic region under the Atlantic influence, characterized by temperate oceanic conditions and relatively homogeneous climatic patterns across the peninsula (Figure 2). The topography is predominantly flat, and elevation varies only slightly among sites, ranging from approximately 10 m to 28 m above sea level. During the sampling period in September 2024, the average temperature recorded at the Pauillac meteorological station was 17.5 °C, with cumulative precipitation of 111 mm, reflecting typical late-season conditions of the region.

Briefly, each estate has independently identified a pool of old candidate vines, sent the scions to a nursery for grafting and propagation, and planted the resulting young vines in their vineyards, in a single trial plot, for further characterization. Sanitary tests were performed throughout the multiplication process to prevent the transmission of regulated grapevine leafroll-associated viruses and grapevine fanleaf virus. Details about each trial are described below.

#### 2.1.1. Chateau Latour

A survey of pre-clonal plots was carried out between 2016 and 2018 to identify old candidate vines of *Cabernet Sauvignon*. Scions from the selected vines were collected, grafted onto rootstock 101-14-Mgt, propagated in a nursery, and eventually planted in a trial plot in 2019. In this plot, each row corresponds to a lineage descending from a single mother vine. We sampled 15 lineages represented by five daughter vines, and their corresponding mother distributed across five plots.

#### 2.1.2. Chateau Lafite

Similarly to Chateau Latour, a survey of old *Cabernet Sauvignon* vines was conducted between 2018 and 2021. A total of 85 vines were selected, propagated in a nursery and grafted onto rootstock 420A. Among these, we sampled 10 older vines, distributed across two distinct terroirs of the estate. As the trial plot was recently implanted, we chose to sample three young vines from plantation surplus deriving from the older ones.

#### 2.1.3. Chateau Leoville Las Cases

In 2019, old *Merlot* vines were selected and classified into three quality categories based on ripening indicators (total acidity, pH, sugars, potential alcohol, total polyphenol index, total anthocyanin content and weight of 100 berries). In this study, we sampled mother vines from two plots, each including three vines of the highest quality and three of the lowest, for a total of 12 mother plants. The daughter vines, planted in 2020 on rootstock 3309C, are located in a plot 20 km north of Chateau Leoville Las Cases. For each of the 12 mother vines, we sampled three corresponding daughter vines.

#### 2.1.4. Chateau Palmer

A survey on one of the pre-clonal *Merlot* plots of the estate resulted in the selection of 84 mother vines. Rootstock 3309C was planted in another plot of the estate in 2018, and two grafting campaigns were carried out in 2021 and 2023 to propagate the selected old vines. We sampled 10 of the mother vines, along with 10 associated daughter vines (five grafted in 2021 and five grafted in 2023).

### 2.2. Sampling

Two compartments—leaves and roots—were analyzed in order to take into account different scion–rootstock combinations. The choice of these compartments was motivated by several considerations. Although recent studies highlight the relevance of shoots and bark for understanding the grapevine microbiome as a single entity [14,15,16], these tissues are more difficult to sample and may weaken the vines, particularly when grafts are recent, as in several of our study sites. While root sampling can also raise similar concerns, it appeared to be more acceptable to vineyard practitioners. Roots were sampled following the protocol described by Biget et al. (2021) [5]. Fine roots were collected at a depth of 5–20 cm, placed individually in labelled sterile bags, and kept refrigerated during transport. In the laboratory, roots were thoroughly rinsed under tap water to remove adhering soil particles and then washed in a diluted Triton X-100 solution to detach surface-associated microorganisms. Samples were then rinsed with sterile Milli-Q water, gently dried on sterile absorbent paper, and subsampled by selecting fragments of fine, living root tissue while avoiding lignified or necrotic roots. The resulting material was transferred to sterile tubes and stored at −20 °C until DNA extraction.

Leaf samples were collected following the protocol described by Agler et al. (2016) [17]. For each vine, five leaves were sampled at comparable height and distance from the trunk. Leaves were first washed in sterile Milli-Q water and a diluted Triton X-100 solution to remove epiphytes. They were then surface-sterilized by brief successive washes in ethanol and sodium hypochlorite, followed by three rinses in sterile Milli-Q water to remove sterilizing agents. Leaf tissues were then dried on sterile absorbent paper, cut into small fragments, transferred to sterile microtubes, and stored at −20 °C until DNA extraction.

In addition, soil samples were collected to characterize the microbiological terroir of the vineyard plots and to establish the soil–root–leaf continuum. Soil sampling followed the standardized protocol developed in the EcoFINDERS project [18]. For each vine, five subsamples were collected at approximately 20 cm depth (one central point and four points located 2 m away along the cardinal directions). Subsamples were pooled, manually cleared of plant debris and stones, homogenized, and sieved through a 4 mm mesh to obtain a representative composite sample. Approximately 250 g of homogenized soil was then placed in sterile bags and stored at −20 °C until DNA extraction.

### 2.3. Molecular Biology

DNA from plant tissue fragments was extracted by the Gentyane platform (UMR 1095 GDEC) using a Sbeadex™ kit (LGC, Teddington, UK) automated on an oKtopur™ robot (LGC). DNA quantification was performed with Hoechst 33258 dye using an Infinite^®^ M1000 plate reader (TECAN, Männedorf, Switzerland). DNA from soil samples was extracted by the GenoSol platform (UMR 1347 Agroécologie) following the protocol described by Ranjard et al. (2003) [19]. Sequencing and preliminary processing steps were carried out at the Genomics and Transcriptomics Platform of Bordeaux (UMR 1202 BIOGECO) for all samples.

Two sequencing targets (fungi and bacteria) were considered using a metabarcoding approach. Fungal communities were analyzed through PCR amplification of an 18S rRNA fragment using the fungal-specific primers NS22b (5′-AATTAAGCAGACAAATCACT-3′) and SSU817 (5′-TTAGCATGGAATAATRRAATAGGA-3′) [20]. Similarly, bacterial communities were analyzed by amplifying the V5–V7 regions of the 16S rRNA gene with primers 799F (5′-AACMGGATTAGATACCCKG-3′) and 1223R (5′-CCATTGTAGTACGTGTGTA-3′) [10]. These primer sets are commonly used in grapevine studies because they avoid amplification of plant DNA while providing broad coverage due to the targeted 18S and 16S rRNA regions [5,21].

PCR products were purified with magnetic beads, quantified by fluorometry, and normalized. A second PCR (Illumina multiplexing) was then performed to produce the sequencing library. This library was purified, quantified, sequenced in short read pairs of 2 × 250 pb on an Illumina MiSeq platform, resulting in 118.1 M reads for 18S DNA and 336.8 M reads for 16S DNA.

### 2.4. Bio-Informatics

Sequence data were processed using the FROGS 4.1 pipeline in Galaxy [22]. FROGS standard protocol was followed for pre-process (including merging reads, removal of primers and amplicon smaller than 350 filtering), then swarm clustering was performed as recommended with a distance of 1 and *fastidious* option. Chimera was removed and a filter was applied on the resulting sequence abundancy to keep clusters with at least 0.005% of all sequences as suggested by Bokulich et al. [23]. This resulted in 860 ASVs representing 73.4% of total abundancy for 18S marker gene and 1119 ASVs corresponding to 78.4% of sequences for 16S marker gene. These initial steps were followed by taxonomic affiliation using BLAST 2.10 on SILVA 138.1 18S and 16S databases [24], with a 95% identity and 95% coverage thresholds. This final step resulted in 734 ASVs for 18S marker (43 ASVs removed corresponding to 2.4% of sequences) and 1094 ASV for 16S marker (25 ASVs removed corresponding to 1.3% of sequences).

### 2.5. Statistical Analysis

All statistical analyses were conducted in R v.4.2.2 [25] using *phyloseq* [26], *microeco* [27], *MicEco* [28] and *NST* [29] packages for microbial community analysis. Graphs were generated with *ggplot2* [30], *ggh4x* [31] and *ggnested* [32] packages. One of the tested variables, Chateau (also referred as site effect), is a group of parameters that cannot be deconvoluted in our experimental design, including vineyard’s geographical location, agricultural practices, cultivar and rootstock.

### 2.6. Testing H1: Generation (Mother or Daughter Vine) Influences Microbial Community Structure and Composition

To understand which factors shape microbial communities, we performed a principal coordinates analysis (PCoA) on Bray–Curtis distance, followed by a PERMANOVA. We then studied communities’ structure by calculating alpha-diversity indices (Chao1 richness, ACE, Shannon, Simpson, Inverse Simpson, Fisher, and Pielou evenness). Differences in alpha-diversity between generations (old vines vs. progeny) were tested using Wilcoxon test. To further refine these results, we complemented the previous analysis with an LEfSE (Linear Discriminant Analysis Effect Size) approach [33] on the relative abundance data at the family level. This approach combines non-parametric statistical testing with linear discriminant analysis to detect taxa whose consistent differences explain the greatest proportion of variance among groups.

### 2.7. Testing H2: Daughter Vines Host Microbial Communities That Are More Phylogenetically Similar to Their Corresponding Mother Vine than to Unrelated Vines

To explore whether microbial communities of offspring resemble those of their mother vines, we conducted a hierarchical clustering analysis using Bray–Curtis distance. This approach allows us to examine how closely related the microbial assemblages are within each vineyard and to evaluate the degree of conservation or divergence between generations across tissue types. Clustering was conducted independently for each Chateau and tissue type to account for vineyard-specific and compartment-specific effects. The resulting dendrograms allowed us to visually assess whether daughter plants grouped preferentially with their mother vine, as expected under a scenario of partial microbiome inheritance.

We also quantified the strength of mother–offspring similarities by computing Bray–Curtis distances for each paired mother–daughter sample. Pairs were classified as clustered when their Bray–Curtis distance fell within the first quartile of the overall distance distribution, a statistic-driven threshold commonly used to identify the closest associations in the absence of an absolute biological cut-off. For each Chateau, we then calculated the proportion of clustered mother–daughter pairs and visualized these proportions using bar plots, allowing a quantitative comparison of clustering frequency across vineyards and tissue types.

### 2.8. Testing H3: The Assembly of Microbial Communities Between Mother Vines and Their Offspring Is Primarily Driven by Deterministic Processes

We evaluated whether mother vines exert a detectable selective influence on the assembly of their daughter–plant microbiomes with the Beta Nearest Taxon Index (βNTI) [34] for all pairwise comparisons within each mother–daughter family. To determine whether the observed βNTI values reflected a non-random, potentially directional influence of the mother plant, we computed a permutation-based significance test. In this procedure, the assignment between mothers and daughters was repeatedly randomized to generate a null distribution of βNTI values representing stochastic associations. The observed βNTI differences were subsequently compared to this null distribution to assess whether phylogenetic turnover between actual mother–daughter pairs was significantly higher or lower than expected by chance. The resulting Z-scores allow us to test whether vertical transmission or host filtering leads daughters to retain phylogenetically non-random subsets of their mother’s microbiome, or conversely, whether mother–daughter relationships are indistinguishable from stochastic assembly dynamics.

We complemented the βNTI-based assessment of phylogenetic turnover, by calculating the Normalized Stochasticity Ratio (NST) [35] for each mother–daughter family in order to evaluate the relative contribution of stochastic versus deterministic processes shaping microbial community assembly. To assess whether the observed NST values reflect a true mother-driven deterministic filtering (NST < 50%) or random assembly dynamics (NST > 50%) such as ecological drift or random dispersal, we performed a permutation-based significance test analogous to the one used for βNTI. In this test, the associations between mothers and daughters were repeatedly randomized to generate a null distribution of NST values corresponding to stochastic pairings. By comparing the empirical NST values to this null expectation with a Z-score, we assessed whether real mother–daughter pairs show a significantly more deterministic (or more stochastic) assembly pattern than it would occur by chance. This analytical framework enables us to test whether daughter plants tend to inherit a deterministically filtered microbial subset reflecting maternal influence, or whether their microbiomes assemble predominantly through stochastic processes independent of the mother plant.

## 3. Results

### 3.1. H1: Sample Type and Chateau Have More Influence on Community Composition and Structure than Generation

**Figure 3:**
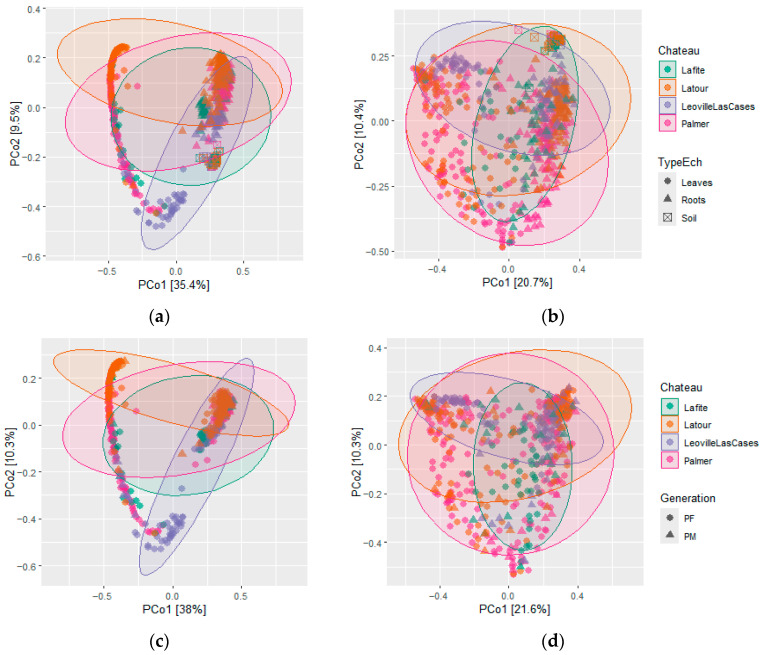
Distribution of the microbial communities across Chateau, sample type (**a**,**b**) and generations (**c**,**d**). PCoA of bacterial (**a**,**c**) and fungal (**b**,**d**) communities, where samples originating from the same vineyard share the same colour. Symbols respectively indicate the sample type (circles for leaves, triangles for roots, and squares for soil) or plant generation (circles for daughter vines PF, and triangles for mother vines PM).

Bacterial communities display clear differences depending on sampling compartment, with roots, leaves and soil communities forming two distinct groups on the PCoA ordination (Figure 3). Within each compartment, an additional structuring by vineyard tends to appear. Fungal communities show the same pattern as for bacterial community, but with stronger overlaps. However, there is no clear separation observed between mother plants and their offspring at this scale for either bacterial or fungal communities. These visual trends suggest that plant compartment and vineyard origin exert stronger effects on bacterial community composition than grapevine generation.

This pattern is further evaluated by PERMANOVA performed for the Chateau factors, sample type, generation, and their combinations, which all had a significant effect (Table S1). When considered separately, sample type has the highest R^2^ (R^2^ = 0.35 and R^2^ = 0.16, respectively, for bacteria and fungi), while Chateau explains 10% of variability for bacteria and 7.3% for fungi. Generation also has a significant impact, but of lower extent (R^2^ = 0.005 and R^2^ = 0.01, respectively, for bacteria and fungi). When looking closer at PERMANOVA results for PM/PF comparisons according to sample site and tissues, some exceptions are to be noted (Table S2). While all comparisons show a significant effect, bacterial communities of leaves in Chateau Latour and in Chateau Palmer are not significantly different, as well as fungal communities for leaves at Chateau Latour and roots at Chateau Palmer.

**Figure 4:**
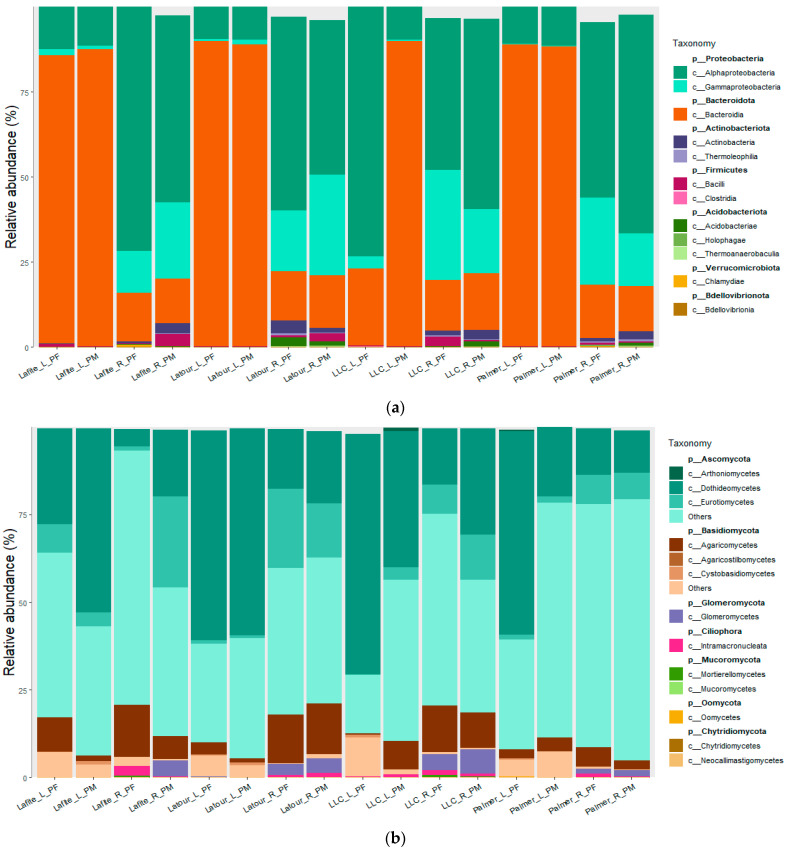
Microbial community profiles of fungal (**a**) and bacterial (**b**) classes, according to vineyard, sample type and generation. Names are coded as following: vineyard (Lafite, Latour, LLC = Leoville las Cases, Palmer), sample type (L = leaves; R = roots) and generation (PF = daughter lines; PM = mother plants).

Bar plots of class-relative abundance also reveal different community profiles (Figure 4). Fungal communities are characterized by a high proportion of Ascomycota, with the Arthoniomycetes class being dominant in leaves, while roots are comparatively enriched in Agaricomycetes (Basidiomycota). Subtle differences appear when comparing PM and PF, mainly regarding relative abundances of Ascomycota and Basidiomycota, and also with specific phyla in PF like Mucoromycota. Bacterial communities are dominated by Bacteroidota in leaves, while Proteobacteria are more abundant in roots. As for fungi, differences in PM and PF appear when considering less abundant classes like Actinobacteria, Bacili or Acidobacteria, especially in roots.

Regarding community structures, Wilcoxon tests on alpha-diversity indices show distinct patterns depending on the sampling site (Table S3). Significant differences between mother vines (PM) and daughter vines (PF) were mainly observed at Chateau Lafite and Chateau Leoville Las Cases, whereas communities from Chateau Latour and Chateau Palmer showed comparable alpha-diversity between generations. The only exception was bacterial richness in roots at Chateau Palmer, which was significantly higher in PM. At Chateau Lafite and Chateau Leoville Las Cases, differences between generations were identified in both compartments, although their direction varied. In leaves, bacterial and fungal communities showed similar richness between generations at Chateau Lafite but higher diversity in PF, whereas both richness and diversity were higher in PF at Chateau Leoville Las Cases. In roots, the opposite pattern was generally observed: at Chateau Lafite, both bacterial and fungal communities displayed higher richness and diversity in PM. At Chateau Leoville Las Cases, this trend was detected only for bacterial communities, which were significantly richer and more diverse in PM.

**Figure 5:**
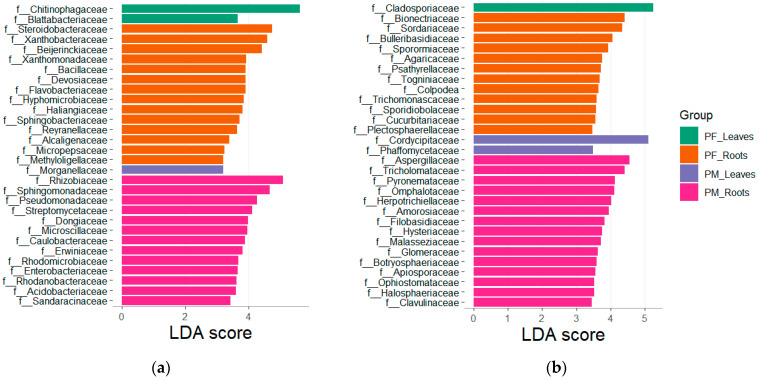
Microbial families significantly enriched depending on sample type and generation. Fungal (**a**) and bacterial (**b**) families with the 30 highest LDA scores are coloured as following: green for leaves of daughter lines, orange for roots of daughter lines, purple for leaves of mother vines, and pink for roots of mother vines.

At a larger scale, we finally targeted biomarkers of PM and PF for both leaves and roots with an LEfSE analysis and represented the highest 30 LDA score at the family rank (Figure 5). Chitinophagaceae, Blattabacteriaceae and Cladosporiaceae are preferentially associated with PF leaves, while Morganellaceae, Cordycipitaceae and Phaffomycetaceae are preferentially associated with those of PM. Regarding roots, a higher number of taxa are associated both for PM and PF (i.e., Steroidobacteraceae, Xanthobacteraceae, Bionectriaceae, Sordariaceae for PF, and Rhizobacter, Sphingomonadaceae, Aspergillaceae and Tricholomataceae in PM). Taxa that are enriched in progeny potentially reflect early-colonization processes, persistent nursery effects, or environmental filtering during establishment. Those enriched in mother vines are rather possibly associated with long-term vineyard conditions, accumulated exposures, or stable host–microbe associations. These taxa being strongly associated with PM may be less likely to be passed on to their massal offspring.

Overall, H1 hypothesis is partially supported: generation influences microbial community structure and composition, but this effect is modest, compared to tissue and site effects.

### 3.2. H2: Hierarchical Clustering Reveals Weak and Context-Dependent Mother–Daughter Resemblance

Hierarchical clustering based on Bray–Curtis dissimilarities provides a visualization of microbial resemblance between mother vines and their progeny and reveals contrasting patterns across vineyards. Two main configurations emerged and were applied to both bacterial and fungal communities, independently of the sampled tissue.

**Figure 6:**
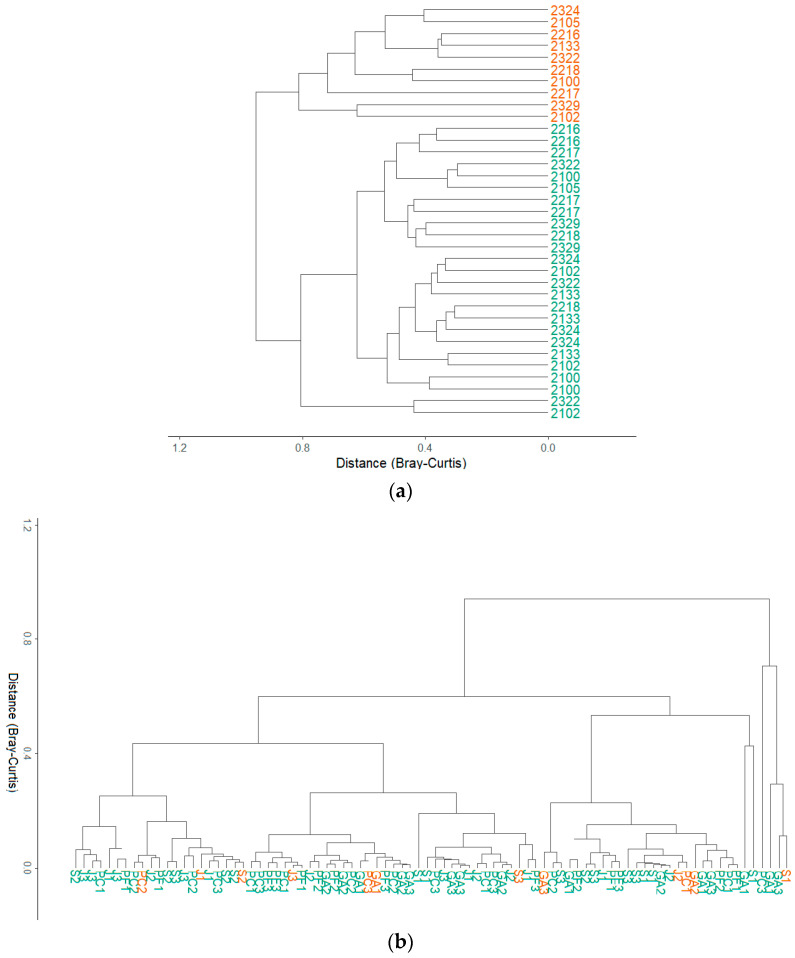
Hierarchical clustering based on Bray–Curtis dissimilarities of mother vines and their progeny. Samples belonging to the same lineage share the same name; mother plants are coloured in orange, offspring in green. Dendrogram (**a**) represents bacterial community in leaves at Chateau Lafite; dendrogram (**b**) represents bacterial community of leaves at Chateau Latour. See Supplementary Data S1 for other figures.

**Figure 7:**
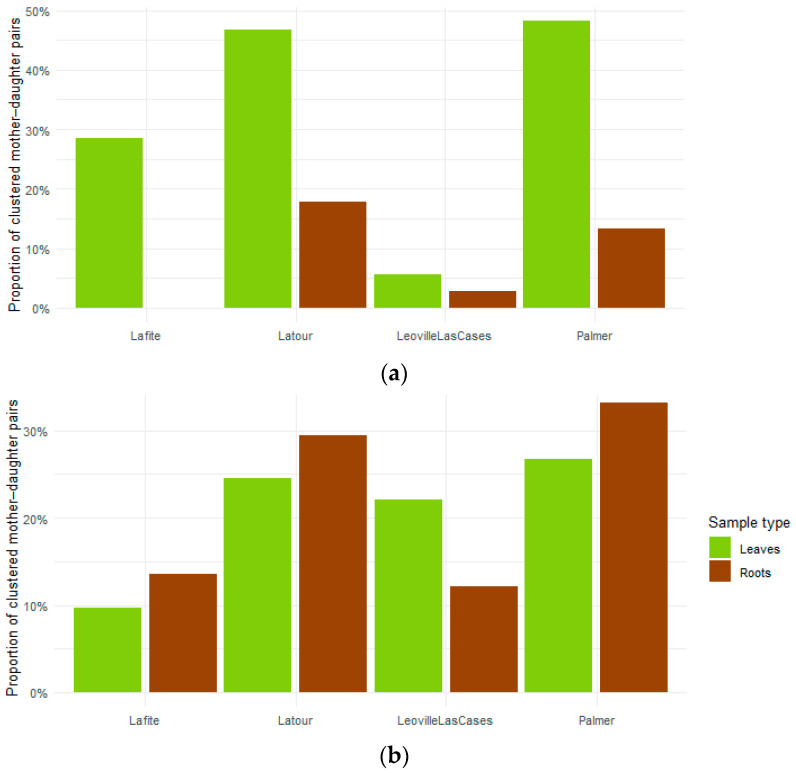
Frequency of mother–daughter clustering based on Bray–Curtis dissimilarities. Pairs are considered clustered when falling into the first quartile. Bacterial communities are represented in figure (**a**) and fungal communities in figure (**b**).

At Chateau Leoville Las Cases and Chateau Lafite, samples tended to cluster primarily by generation rather than by maternal lineage (Figure 6a). In these vineyards, mother vines (PM) formed distinct groups from progeny (PF), while daughter vines did not show a clear tendency to cluster by lineage, indicating that shared maternal origin did not strongly influence microbial community. These observations are supported by the lower proportions of clustered mother–daughter pairs quantified from Bray–Curtis distances in these two estates (Figure 7) (complete dendrograms in Supplementary File S1).

On the other side, Chateau Latour and Chateau Palmer displayed a different pattern (Figure 6b). In these vineyards, a limited number of daughter vines pair closely together with their corresponding mother (for example lineage S2, J1 or PC2), suggesting higher microbial similarity within specific lineages. This trend was confirmed by higher proportions of clustered mother–daughter pairs compared to Chateau Lafite and Chateau Leoville Las Cases (Figure 7). These proportions were particularly elevated in leaf-associated communities for bacteria, whereas fungal communities showed lower but still consistent levels of clustering across tissues. In a few cases, small groups of progenies from the same lineage also clustered together while not being associated with their mother. However, these patterns remained infrequent, and most daughter vines clustered independently of their mother vine and family affiliation. Overall, clustering by lineage was not a dominant feature of the dendrograms (File S1).

Taken together, these results indicate that microbial communities rarely cluster strictly according to mother–daughter relationships, and that lineage-specific similarity, when present, represents a minority of cases. Thus, hierarchical clustering and distance-based quantification provide limited support for H2 hypothesis, suggesting weak and inconsistent microbial resemblance between mother vines and their progeny. While lineage-specific clustering can occasionally be detected and quantified, environmental conditions and generation (mother vs. daughter) emerge as stronger determinants of microbial community structure than maternal relatedness.

### 3.3. H3: Mother-Offspring Microbial Assemblies Reflect a Balance Between Deterministic and Stochastic Processes

**Figure 8:**
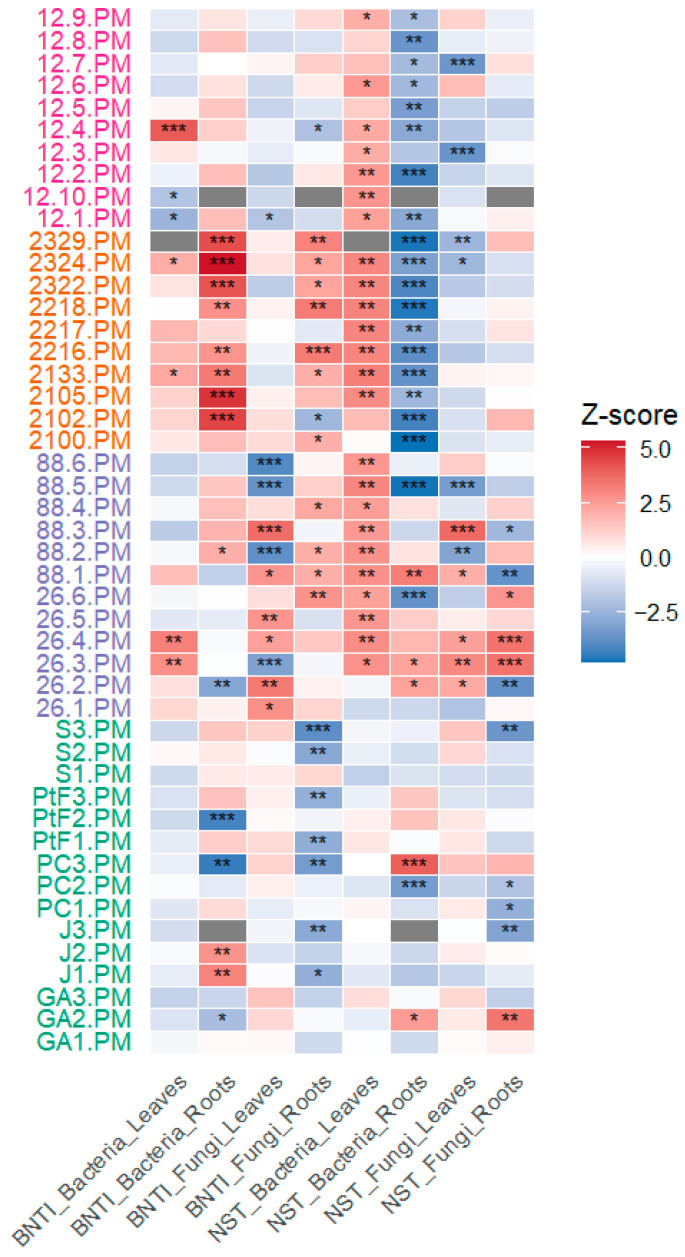
Deviations from null expectations of βNTI and NST computed for fungal and bacterial communities in leaves and roots. Names of mother plants are coloured according to vineyard of origin: Chateau Palmer in pink, Chateau Lafite in orange, Chateau Leoville las Cases in purple and Chateau Latour in green. Asterisks indicate statistically significant deviations from null expectations based on permutation tests: * for *p*-value < 0.05; ** for *p*-value < 0.01; *** for *p*-value < 0.001. Significant high absolute Z-scores indicate non-random assembly while Z-score around 0 is indistinguishable from stochastic assembly dynamics.

The βNTI and NST analyses (Figure 8) reveal a strong heterogeneity in microbial community assembly processes between mother vines and their offspring. Significant deviations from null expectations are detected in numerous mother–daughter comparisons; however, these signals are unevenly distributed across vineyards, tissues, and microbial kingdoms, indicating that deterministic assembly processes are neither universal nor consistent across contexts.

When accounting for vineyard origin, site-dependent patterns emerge. Mother vines from Chateau Lafite (orange labels) exhibit the highest frequency of statistically significant Z-scores, particularly for bacterial communities associated with roots. In this vineyard, multiple mother vines show significant βNTI values, most often with positive Z-scores, together with significant NST values indicative of non-stochastic assembly. These results indicate that phylogenetic turnover between mothers and daughters at Chateau Lafite frequently departs from random expectations, reflecting strong deterministic processes. Importantly, the predominance of positive βNTI Z-scores suggests that this determinism is primarily associated with phylogenetic divergence rather than increased similarity between generations.

Mother vines from Chateau Leoville Las Cases (purple labels) also show numerous significant Z-scores for both βNTI and NST, although these signals are more heterogeneous in direction and tissue specificity. Significant deviations from stochastic assembly are observed in both bacterial and fungal communities, particularly in roots, but are interspersed with non-significant comparisons. This pattern suggests that deterministic processes are present but depend on lineage and local context, with no consistent tendency toward either phylogenetic conservation or divergence.

In contrast, mother vines from Chateau Latour (green labels) display the lowest frequency of significant Z-scores across all indices. Most βNTI and NST values fall within the range expected under stochastic assembly, indicating that microbial community assembly between generations in this vineyard is largely governed by stochastic processes such as dispersal and ecological drift.

Finally, Chateau Palmer (pink labels) stands in an intermediate position between these two contrasted patterns. Although some mother vines display statistically significant βNTI or NST Z-scores, many comparisons remain non-significant, suggesting that deterministic influences on community assembly are sporadic rather than pervasive at this site. Compared to Chateau Latour, Chateau Palmer shows a higher frequency of detectable deterministic signals, yet these signals are clearly weaker and less systematic than those observed at Chateau Lafite and Chateau Leoville Las Cases. Overall, Chateau Palmer represents an in-between configuration where microbial assembly across generations reflects a balance of stochastic dynamics and localized deterministic filtering, rather than a strong or uniform maternal influence.

Across all vineyards, significant Z-scores are more frequently observed in root-associated communities than in leaves, and more often for bacterial communities than for fungal ones. Notably, significant βNTI Z-scores include both positive and negative values, indicating that deterministic assembly does not consistently result in increased phylogenetic similarity between mothers and daughters. Instead, significant deviations reflect context-dependent phylogenetic filtering or lineage replacement rather than systematic vertical transmission.

Overall, the combined βNTI and NST results indicate that deterministic processes can influence microbial community assembly between mother vines and their offspring, but that this influence is highly dependent on vineyard context (i.e., geographical location, cultural practices, scion genotype and rootstock), tissue type, and microbial kingdom. These findings provide partial support for H3 hypothesis, while emphasizing that deterministic assembly is not a dominant or universal mechanism, but rather one component of a broader assembly continuum shaped by strong local environmental filters.

## 4. Discussion

### 4.1. Environmental Context Outweighs Generation Effects in Grapevine Microbiome Assembly

Across all analyses, microbial community composition and structure were primarily driven by plant compartment and vineyard, whereas generation (mother or daughter vine) played a comparatively minor role. Both bacterial and fungal communities showed strong compartmentalization, with additional structuring by Chateau, highlighting the dominant influence of local environmental conditions, management practices, cultivar and rootstock. This pattern is consistent with numerous studies demonstrating that soil properties, microclimate, and plant compartment act as primary filters of plant-associated microbiomes [36,37,38]. Although generation had a statistically significant effect, its explanatory power remained limited relative to these factors, indicating that grapevine-associated microbial communities are only weakly conserved across clonal generations.

These results suggest that, following propagation and planting, microbial communities are largely reassembled under local environmental constraints. Similar conclusions have been reached in grapevine and other perennial systems, where microbiome composition shows high plasticity and rapid adjustment to external ecological drivers [5,17]. Together, these findings support a view of the grapevine microbiome as an environmentally structured system rather than a strictly inherited trait.

### 4.2. Limited and Context-Dependent Microbial Resemblance Between Mother and Daughter Vines

Hierarchical clustering analyses provided limited support for lineage-driven microbial similarity. In most cases, daughter vines did not cluster with their corresponding mother, nor did they consistently group by family, indicating that shared maternal origin alone does not strongly structure microbial communities. Similar patterns have been reported in clonal and seed-propagated plants, where vertical transmission of microbiota is partial [10] and overridden by environmental acquisition [39].

At vineyards where mother and daughter vines experienced contrasting conditions (i.e., nursery versus established vineyard soils at Chateau Lafite or spatially distinct plots at Chateau Leoville las Cases), microbial communities clustered primarily by generation, suggesting environmental discontinuity as the dominant driver. Conversely, for sites where environmental conditions were more similar between mother and daughter vines (mother vines’ plots and trial plot in the same Chateau), occasional lineage-level clustering was observed. However, these cases remained rare and did not constitute a dominant pattern. Overall, these findings indicate that microbial inheritance between mother vines and their progeny is weak and inconsistent, and that apparent similarity likely reflects environmental continuity or convergence rather than systematic vertical transmission.

### 4.3. Environmental Transitions During Propagation as a Potential “Nursery Filter”

The limited microbial resemblance observed between mother vines and their progeny may also reflect environmental transitions occurring during the propagation process. In mass selection, scions are collected from old vines, grafted and cultivated in nurseries before being replanted in vineyards. These transitions may act as strong ecological filters on plant-associated microbiomes. During nursery production, microbial communities associated with scions could be partially lost, replaced, or reassembled under the influence of the nursery environment and grafting. Transplantation into vineyard soils may further reshape microbial communities through local environmental filtering and microbial recruitment from surrounding soil reservoirs.

This “nursery filter” hypothesis provides an alternative explanation for intergenerational microbiome differences. Rather than reflecting weak microbial inheritance, the observed divergence between mother and daughter microbiomes may result from successive ecological filtering events during propagation and establishment. In this context, microbial inheritance may be partly masked by environmental reassembly processes occurring during plant propagation. Understanding how nursery conditions influence early microbiome establishment is an interesting perspective for future research in perennial crop systems.

### 4.4. Deterministic Assembly Processes Operate but Do Not Imply Microbial Inheritance

Process-based analyses using βNTI and NST revealed that deterministic forces can influence microbial assembly between generations, but in a heterogeneous and site-dependent manner. Significant deterministic signals were detected in several comparisons, particularly in root-associated bacterial communities, yet these signals were unevenly distributed across vineyards, tissues, and microbial kingdoms. Such heterogeneity aligns with current ecological theory, which predicts that deterministic and stochastic processes jointly contribute to microbial community assembly, with their relative importance varying across spatial and environmental gradients [34,35,40].

Importantly, deterministic assembly did not consistently result in increased phylogenetic similarity between mother and daughter vines. Both positive and negative βNTI deviations were observed, indicating selective filtering or lineage replacement rather than straightforward conservation of maternal microbiota. Our results suggest that deterministic processes may drive divergence rather than inheritance, and that stochastic processes such as dispersal and ecological drift remain major contributors to microbial assembly, particularly where environmental contrasts are limited.

### 4.5. Avoiding an Adaptive Bias in Interpreting Plant–Microorganism Relationships

Our results highlight a potential adaptive bias when thinking about plant–microorganism interactions, especially in clonal systems. While co-evolutionary and inheritance-based frameworks are central to population genetics, they may be less appropriate when considering microbial communities characterized by rapid turnover, high dispersal, and strong environmental sensitivity. Conceptual critiques of the holobiont and hologenome concepts have indeed emphasized that microbial associations are not necessarily stable, adaptive, or heritable across generations [41,42].

The weak and context-dependent microbial resemblance observed here supports a more ecological interpretation, in which many microbial patterns emerge from neutral dynamics, environmental filtering, and historical contingency rather than from host-driven selection alone [43]. In our case, this perspective cautions against overinterpreting microbial similarity as evidence of adaptive inheritance.

However, the limited microbial inheritance detected in this study does not diminish the functional importance of microbiomes for grapevine performance. Rather, it underscores that microbial communities represent only one component of a broader, multifactorial inheritance system in mass selection. Genetic variation, grafting practices, rootstock identity, and propagation conditions, as well as other factors, all contribute to trait transmission across generations [7,44].

The limited evidence for consistent microbial inheritance observed in this study also raises questions about the potential role of active microbiome management strategies, such as microbial inoculations or biocontrol applications, which are increasingly explored to enhance plant resilience. However, the strong environmental filtering and stochastic dynamics observed in microbial assembly suggest that introduced microorganisms may not persist within established microbial networks, which is consistent with a growing number of studies highlighting the difficulties of maintaining exogen communities in field conditions [45,46]. In this perspective, managing environmental conditions and cultivation practices that favour beneficial microbial recruitment may be a more robust and effective strategy.

### 4.6. Importance of the In Situ Framework and Implications for Sustainable Viticulture

A key feature of this study is its entirely in situ design, across multiple vineyards, tissues, and contrasting environmental contexts. While such ecological realism inevitably introduces variability and background noise, it also provides a more accurate representation of microbial assembly under real viticultural conditions. Although several generation-related effects were statistically significant, their effect sizes remained relatively small, indicating that differences between mother and daughter vines are subtle rather than dominant drivers of community variation. Despite this limitation and the complex study design, we consistently detected statistically significant patterns across a large number of samples, suggesting that the observed signals are biologically meaningful rather than artefactual.

By integrating community-level patterns with assembly process metrics, this study offers a realistic assessment of microbial inheritance in a perennial crop system. Rather than invalidating mass selection, our results suggest that it operates within a subtle and environmentally modulated network of plant–microorganism interactions. This nuanced view aligns with sustainability-oriented approaches that prioritize system-level resilience and ecological plasticity over fixed biological traits.

## 5. Conclusions

This study shows that microbial community structure in grapevine is primarily shaped by plant compartment and vineyard context, while generation effects between mother vines and their progeny remain weak and inconsistent. Across bacterial and fungal communities, microbial resemblance between mother and daughter vines are rarely conserved and are strongly dependent on environmental continuity rather than lineage. Process-based analyses further indicate that microbial assembly reflects a balance between deterministic and stochastic forces, without evidence for systematic microbial inheritance through clonal propagation.

Together, our findings challenge an overly adaptive or inheritance-centred interpretation of plant–microorganism relationships and highlight the high ecological plasticity of grapevine-associated microbial communities. From a sustainability perspective, they suggest that environmental management and cultivation practices are likely to play a more decisive role than propagation material alone in shaping vineyard microbiomes. Integrating microbial ecology with genetic, epigenetic, and agronomic approaches will be essential to better understand how multiple inheritance components interact in perennial cropping systems.

## Data Availability

Absolute abundance data for fungal and bacterial communities are available from Recherche Data Gouv under the DOI [https://doi.org/10.57745/C2KWFV].

## Supplementary Materials

The following supporting information can be downloaded at https://www.mdpi.com/article/10.3390/microorganisms14030622/s1, File S1: Bray–Curtis dendrograms; Table S1: Permanova results for community comparisons of PM/PF; Table S2: Decomposition of permanova results for community comparisons of PM/PF; Table S3: Wilcoxon test results on diversity indexes, comparing PM vs. PF for each combination of vineyard and sample type.
